# Analytical validation, sample stability, and clinical evaluation of a new high-sensitivity cardiac troponin I immunoassay for use in dogs, with comparison to a previous ultrasensitive assay

**DOI:** 10.1371/journal.pone.0288801

**Published:** 2023-07-18

**Authors:** Sonya Wesselowski, Jonathan Lidbury, Ashley B. Saunders, Sonya G. Gordon, Jan S. Suchodolski, Joerg M. Steiner

**Affiliations:** Department of Small Animal Clinical Sciences, School of Veterinary Medicine and Biomedical Sciences, Texas A&M University, College Station, TX, United States of America; Second Xiangya Hospital, CHINA

## Abstract

Cardiac troponin I (cTnI) is considered the gold standard biomarker for myocardial injury and shows a high degree of homology between humans and dogs. The ADVIA Centaur XP High-Sensitivity Troponin I (AC-cTnI-HS) assay has been validated for use in humans but not dogs. The study objectives were to analytically validate the AC-cTnI-HS assay in dogs, to assess correlation between the AC-cTnI-HS and a previous ADVIA Centaur TnI-Ultra (AC-cTnI-U) assay, to assess cTnI sample storage stability, and to clinically evaluate the AC-cTnI-HS assay in healthy dogs and dogs with cardiac disease. Canine serum samples were used for analytical validation. Intra- and inter-assay variability, dilutional parallelism, and spiking recovery were assessed. Samples from 196 client-owned dogs were evaluated (healthy dogs (n = 39) or dogs with congenital heart disease (n = 54), myxomatous mitral valve disease (n = 68), dilated cardiomyopathy (n = 15), or myocarditis (n = 20)). Inter- and intra-assay coefficient of variation (%CV) was between 2.8–41.4% and 3.8–30.2%, respectively, with pools with concentrations >20 pg/mL all having %CVs <10%. The observed to expected ratios for dilutional parallelism and spiking recovery experiments ranged between 92.3 and 266.7.0% and 84.3 and 108%, respectively. A strong correlation between the AC-cTnI-HS and AC-cTnI-U assays was observed (Spearman’s ρ = 0.927), though a proportional bias existed, with AC-cTnI-HS assay concentrations being proportionally lower than AC-cTnI-U assay concentrations. Serum samples stored at -80°C had stable cTnI measurements for up to 2.7 years and after a single freeze-thaw cycle. Healthy dogs and dogs with congenital heart disease had significantly lower cTnI concentrations than dogs in the other three groups. The AC-cTnI-HS assay precisely, reproducibly, and accurately measures cTnI concentrations in dog serum with cTnI concentrations >20 pg/mL.

## Introduction

Cardiac troponin I (cTnI) is considered the gold-standard biomarker for detection of myocardial injury in both humans and dogs [1], with the degree of elevation in cTnI concentrations having been described to be proportional to the degree of myocardial damage [2]. Homology of canine and human cTnI genes is high, supporting the use of commercially available human cTnI analyzers to measure cTnI in dogs after adequate validation [3]. Both standard and ultra-sensitive cTnI assays have been validated [4–7] and studied in dogs, with standard assays constrained by lower limits of detection that are unable to identify lower-level increases in serum cTnI concentrations.

The ADVIA Centaur TnI-Ultra assay, henceforth referred to as AC-cTnI-U, is an ultrasensitive cTnI assay validated for use in the dog (6, 7) that has a detection limit of 0.006–50.0 ng/mL. The AC-cTnI-U assay has been widely used for clinical veterinary patients and in clinical veterinary research [8–23], however, this assay has now been retired and replaced by the ADVIA Centaur XP High-Sensitivity Troponin I assay, henceforth referred to as AC-cTnI-HS. For a troponin assay to be considered high sensitivity, cTnI must be detectable above the limit of detection and below the 99^th^ percentile upper reference limit of normal in at least half of the population, with a coefficient of variation (CV) <10%, as described in a recent review on evaluating analytical performance of troponin immunoassays [24]. The AC-cTnI-HS assay meets criteria to be defined as a high sensitivity assay and has a limit of quantification between 2.5–25,000 pg/mL (ng/L), as stated by the manufacturer. It has been validated in humans and has superior diagnostic ability for identification of cardiac disease [25] and better analytical performance [26] than the previous generation AC-cTnI-U assay. The AC-cTnI-HS has not yet been validated for use in dogs, nor have correlations between the AC-cTnI-U and AC-cTnI-HS assays been reported in dogs.

The objectives of this study were to analytically validate the AC-cTnI-HS assay for use in dogs, to assess the correlation between the AC-cTnI-HS and AC-cTnI-U assays, to assess cTnI sample stability under various storage conditions, and to clinically evaluate the assay in populations of healthy dogs and dogs with cardiac disease.

## Materials and methods

### Analytical validation

Surplus serum samples submitted for diagnostic purposes were used to create pools of serum with AC-cTnI-HS concentrations of approximately 10, 20, 50, 100, 250, and >1000 pg/mL. Intra-assay variability was determined by measuring these six serum pools (mean AC-cTnI-HS concentrations: 10, 15, 49, 104, 290 and 1672 pg/mL) ten times in the same assay run. Inter-assay variability was determined by measuring the same six serum pools (mean AC-cTnI-HS concentrations: 17, 16, 43, 96, 264 and 1467 pg/mL) over ten consecutive days. Linearity was assessed by manual stepwise dilution. Four samples (53, 113, 281, and 1528 pg/mL) were serially diluted (1:2, 1:4, 1:8, 1:16) with sample diluent provided by the assay’s manufacturer. Observed to expected (O/E) ratios were then calculated. To assess assay accuracy by spiking recovery, equal volumes of four serum samples of known concentration (AC-cTnI-HS concentrations: 53, 113, 281, and 1528 pg/mL) were mixed in all possible combinations (A+B, A+C, etc.), and O/E ratios were calculated. The blank was calculated by measuring the sample diluent and the zero calibrator 20 times. The lower limit of the blank was then calculated for each as: mean_blank_ + 1.645 (standard deviation_blank_).

### Method comparison

Paired AC-cTnI-U and AC-cTnI-HS results (pg/mL) from 196 dog serum samples were used for method comparison. Passing-Bablok regression fit and Bland-Altman analysis (AC-cTnI-US–AC-cTnI-U versus mean) were performed for method comparison using an online tool (https://bahar.shinyapps.io/method_compare).

### Sample stability

Cardiac troponin I concentrations were measured on ten serum samples obtained from pet dogs owned by faculty and staff at baseline (on the day the sample was drawn) using the AC-cTnI-HS assay, then frozen at -80°C and thawed for repeat measurement once per week for six consecutive weeks to assess sample degradation after each freeze-thaw cycle.

To assess degradation at refrigerator temperature and room temperature, cTnI concentrations in ten more serum samples obtained from dogs owned by faculty and staff were measured at baseline using the AC-cTnI-HS assay, then five samples were kept in the refrigerator at 4°C and the other five samples were kept at room temperature. These samples were measured daily for up to 14 days or until the sample quantity was insufficient for additional measurements.

Lastly, 30 additional serum sample aliquots previously frozen at -80°C for 590 days (N = 10), 779 days (N = 10), or 1009 days (N = 10) in which cTnI was originally measured in association with another study using the AC-cTnI-U assay [27] were thawed for the first time for repeat measurement of cTnI concentrations using both the AC-cTnI-U assay and the AC-cTnI-HS assay to assess longer-term storage at -80°C and correlation of the two assays.

### Clinical evaluation

Serum samples from 196 dogs were obtained from 1) client-owned dogs presenting for clinical evaluation, 2) client-owned dogs participating in other clinical research projects, or 3) faculty and staff-owned pets. All owners gave informed consent for participation in studies approved by the Texas A&M Institutional Animal Care and Use Committee (IACUC 2020–0193 CA, IACUC 2020–0160 CA, IACUC 2020–0032 CA, IACUC 2020–0184 CA). Dogs were assigned to Group 1 if they were deemed apparently healthy based on a normal history, physical examination, and echocardiogram. Dogs with cardiac disease were assigned to Groups 2–5 based on the results of their echocardiogram and other clinically indicated diagnostic tests, with Group 2 dogs having congenital heart disease, Group 3 dogs having myxomatous mitral valve disease (MMVD), Group 4 dogs having dilated cardiomyopathy (DCM), and Group 5 dogs having myocarditis. All serum samples obtained as part of the clinical evaluation had cTnI concentrations measured with both the AC-cTnI-U and AC-cTnI-HS assays. Dogs with clinical cardiac disease may have been receiving cardiac medications deemed appropriate based on their stage of disease by the attending cardiologist.

### Statistical methods

The distribution of males vs. females between groups was compared using a Chi-Square test. Continuous variables were assessed for normality using Anderson-Darling tests and visual inspection of q-q plots. The data were not normally distributed and were therefore expressed as median (range). Comparisons of AC-cTnI-HS concentrations among groups of dogs were made using a Kruskal-Wallis test, followed by Dunn’s post-test. The AC-cTnI-U concentrations between baseline and long-term storage were compared using Mann-Whitney U tests. Other comparisons of cTnI concentrations between time points were made using Friedman’s tests followed by Dunn’s post-test. The correlation between age and AC-cTnI-HS concentration in healthy dogs was assessed using Spearman’s correlation coefficient. Statistical significance was set at P <0.05. A statistical software package was used for analysis (GraphPad Prism v.8, GraphPad, GraphPad Software, San Diego, CA).

## Results

### Assay validation

The inter-assay %CV ranged between 2.8 and 41.4%, while the intra-assay %CV ranged between 3.8 and 30.2% (Table 1). Given the poor inter- and intra-assay %CV in the serum sample with a cTnI concentration of approximately 10 pg/mL (sample 1), the lowest level of repeatably detectable cTnI using the AC-cTnI-HS assay in clinical samples was considered >20 pg/mL (the approximate concentration of serum sample 2). Serial dilutions showed a mean O/E ratio of 125.7% (standard deviation: 34.2%) and a range of 92.3–266.7% (Table 2). Observed to expected ratios for spiking recovery experiments showed a mean of 95.9% (standard deviation: 9.7%), with a range of 84.3%– 108% (Table 3). The limit of the blank was 4.5 pg/mL for the zero calibrator and 8.5 pg/mL for the sample diluent.

**Table 1:** Reproducibility and precision of the measurement of cardiac troponin I in canine serum using the ADVIA Centaur cardiac troponin I high sensitivity assay.

	Serum sample	Mean (pg/mL)	SD (pg/mL)	CV (%)
Inter-assay variability (n = 10)	1	17	6.9	41.4
	2	16	1.5	9.3
	3	43	4.3	10.0
	4	96	9.2	9.5
	5	264	11.4	4.3
	6	1467	41.3	2.8
Intra-assay variability (n = 10)	1	10	3.0	30.2
	2	15	1.4	9.3
	3	49	3.4	6.9
	4	104	4.6	4.4
	5	290	11.4	3.9
	6	1672	64.2	3.8

CV: coefficient of variation; SD: standard deviation.

**Table 2:** Results for dilutional parallelism of the ADVIA Centaur cardiac troponin I high sensitivity assay for measurement of cardiac troponin I in canine serum.

Serum sample	Dilution	Observed cTnI concentration (pg/mL)	Expected cTnI concentration (pg/mL)	O/E ratio (%)
1	Undiluted	53		
	1:2	24	26	92.3
	1:4	14	13	107.7
	1:8	9	7	128.6
	1:16	8	3	266.7
2	Undiluted	113		
	1:2	60	57	105.3
	1:4	26	28	92.9
	1:8	24	14	171.4
	1:16	10	7	142.9
3	Undiluted	281		
	1:2	148	141	105.0
	1:4	79	70	112.9
	1:8	41	35	117.1
	1:16	23	18	127.8
4	Undiluted	1528		
	1:2	896	764	117.3
	1:4	456	382	119.4
	1:8	222	191	116.2
	1:16	117	96	121.9

Assay linearity was assessed at dilutions of 1:2, 1:4, 1:8, and 1:16. cTnI: cardiac troponin I; O:E: observed to expected ratio.

**Table 3:** Results of spiking recovery for the ADVIA Centaur cardiac troponin I high sensitivity assay for measurement of cardiac troponin I in serum from dogs.

Serum sample combination		Observed cTnI concentration (pg/mL)	Expected cTnI concentration (pg/mL)	O/E ratio (%)
1	+2	70	83	84.3
1	+3	162	167	97.0
1	+4	716	790	90.6
2	+3	174	197	88.3
2	+4	886	820	108.0
3	+4	965	904	106.7

Each sample was spiked into each of the other samples for spiking recovery experiments. cTnI: cardiac troponin I; O:E: observed to expected ratio.

### Method comparison

**Fig 1:**
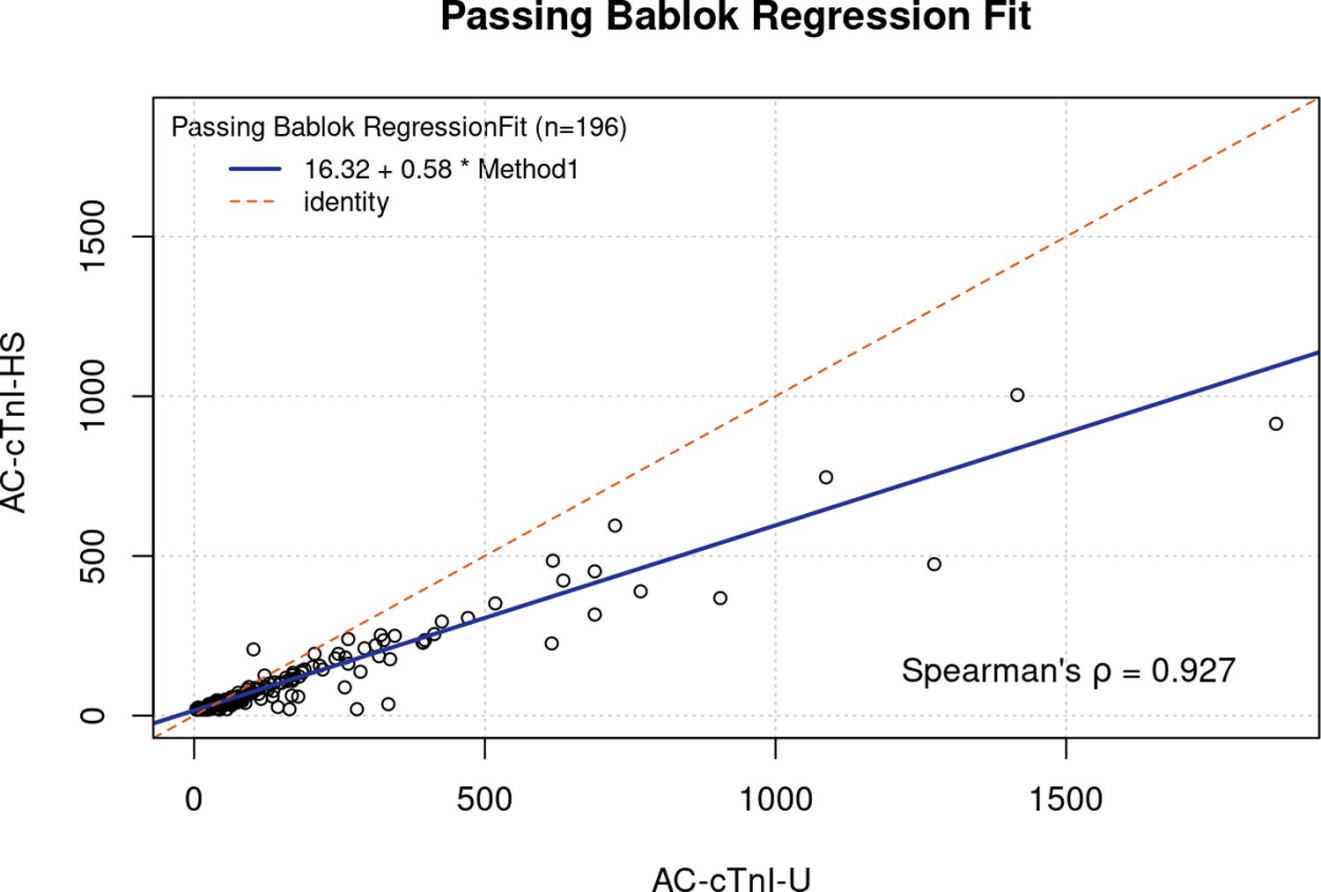
Passing Bablok regression comparison of the ADVIA Centaur XP High-Sensitivity Troponin I assay and the ADVIA Centaur TnI-Ultra assay. Method comparison by Passing Bablok regression revealed a strong correlation between the two assays and a proportional bias whereby the ADVIA Centaur XP High-Sensitivity Troponin I assay measured proportionally lower values than the ADVIA Centaur TnI-Ultra assay.

**Fig 2:**
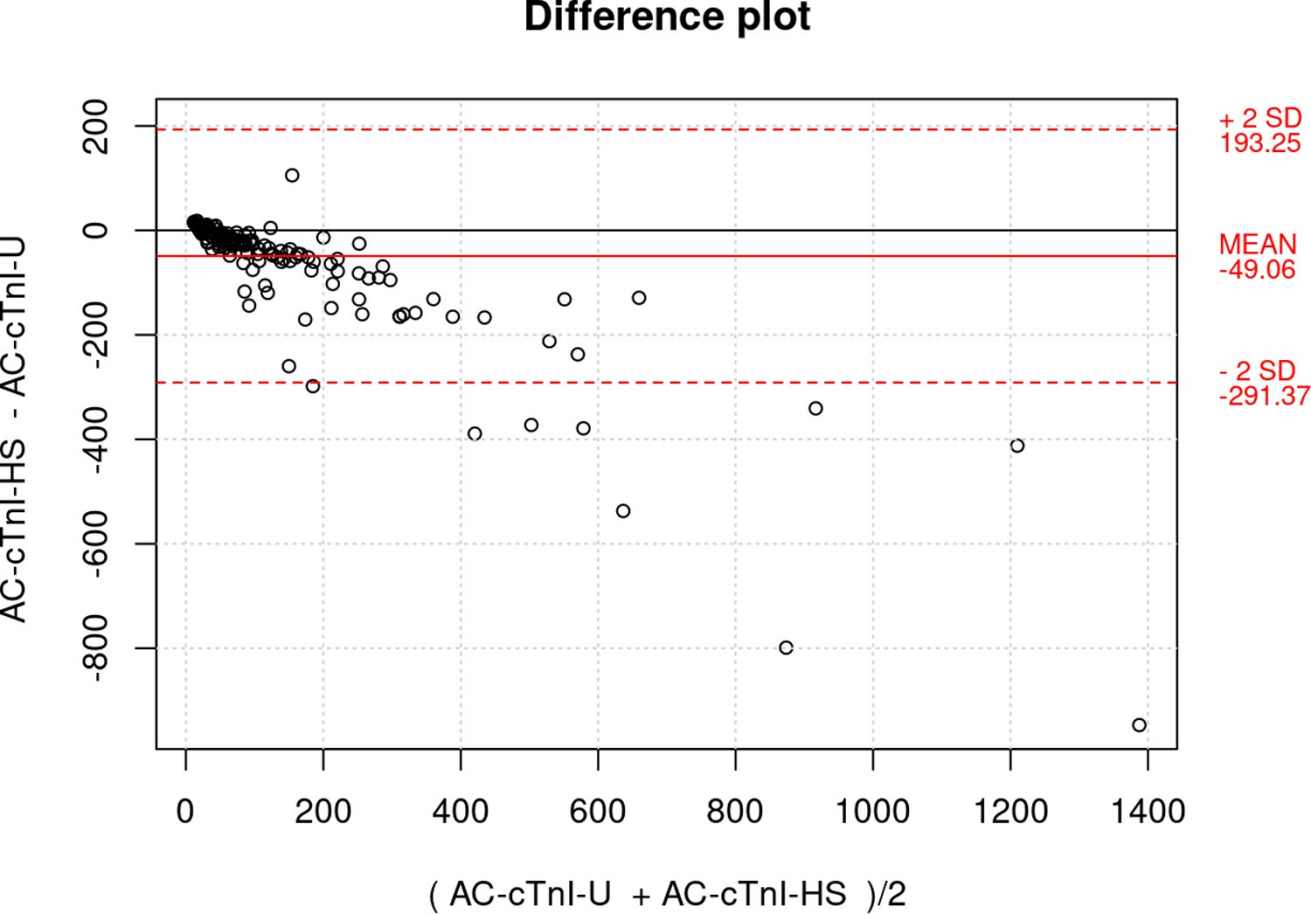
Bland-Altman analysis, comparing the ADVIA Centaur XP High-Sensitivity Troponin I assay and the ADVIA Centaur TnI-Ultra assay. Method comparison with Bland-Altman analysis revealed a mean difference of -49 pg/mL, with limits of agreement between -291 to 193 pg/mL. A proportional bias whereby the ADVIA Centaur XP High-Sensitivity Troponin I assay measured proportionally lower values than the ADVIA Centaur TnI-Ultra assay was noted.

Passing-Bablok regressing analysis revealed a strong correlation between the cTnI results of the AC-cTnI-U and AC-cTnI-HS assays (Spearman’s ρ = 0.927; Fig 1). However, there was evidence of a proportional bias, where the results of the AC-cTnI-HS assay were proportionally lower than those of the AC-cTnI-U assay. Bland-Altman analysis (AC-cTnI-HS–AC-cTnI-U versus mean) is presented in Fig 2, and showed a bias of -49 pg/mL, with 95% limits of agreement, ranging between– 291 to 193 pg/mL. Again, a proportional bias, where the results of the AC-cTnI-HS assay were proportionally lower than those of the AC-cTnI-U assay, was appreciated.

### Sample stability

**Fig 3:**
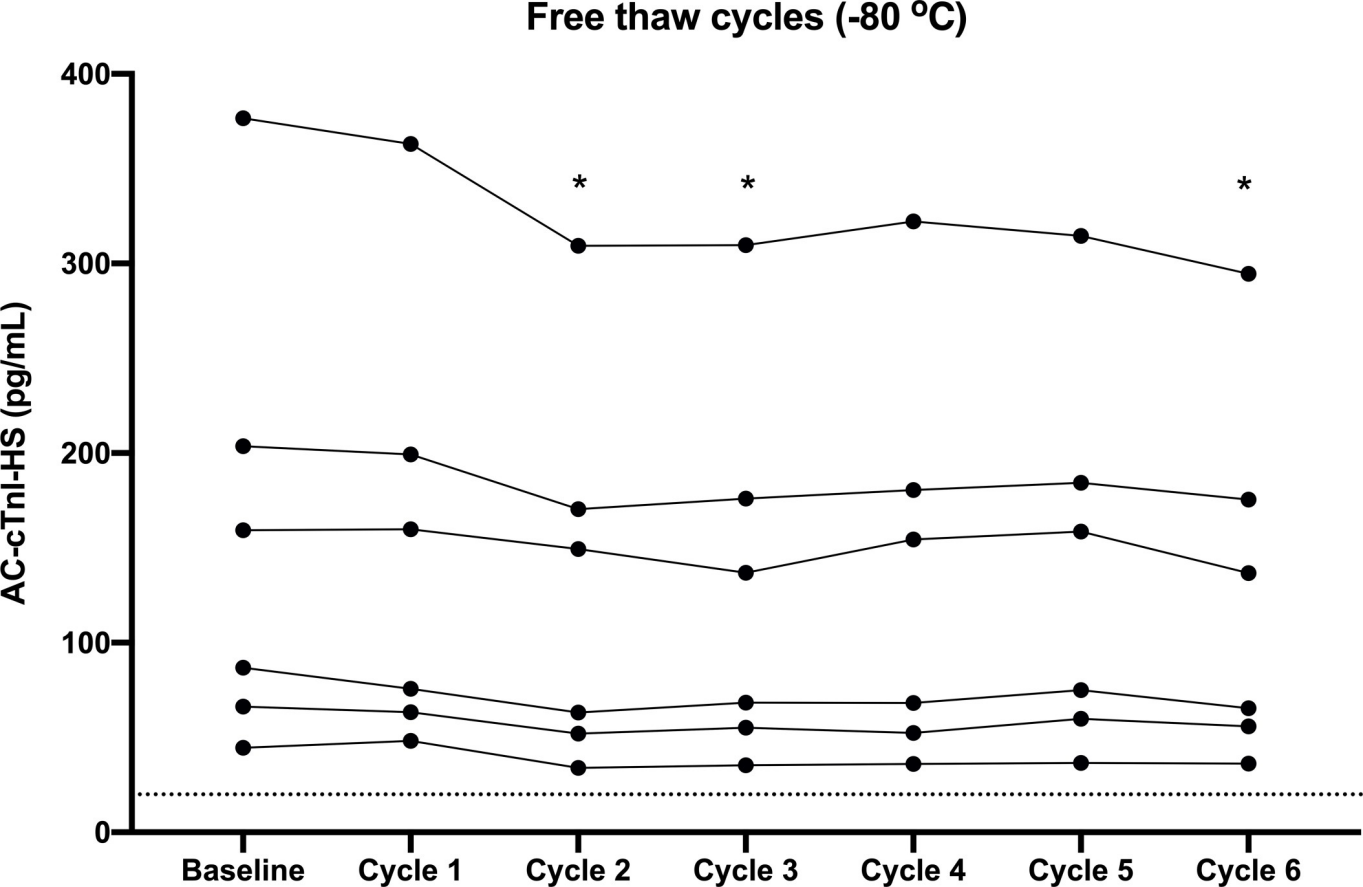
Cardiac troponin I concentrations measured at baseline and after repeated freeze-thaw cycles. Cardiac troponin I measured in serum from dogs utilizing the ADVIA Centaur XP High-Sensitivity Troponin I assay are depicted graphically at baseline and after six consecutive freeze-thaw cycles. Freezer storage was at -80°C. * indicates a significant difference from baseline.

Degradation of cTnI concentrations in serum samples from dogs undergoing freeze-thaw cycles measured with AC-cTnI-HS is presented in Fig 3. Dogs with AC-cTnI-HS values <20 pg/mL at baseline were excluded, leaving six dogs for comparison. The concentration of cTnI was not significantly different from the baseline after one freeze-thaw cycle (P > 0.999). After a second freeze-thaw cycle, however, serum concentrations of cTnl had decreased significantly (P < 0.0002). Serum cTnI concentrations were also lower than baseline at cycles 3 and 6.

**Fig 4:**
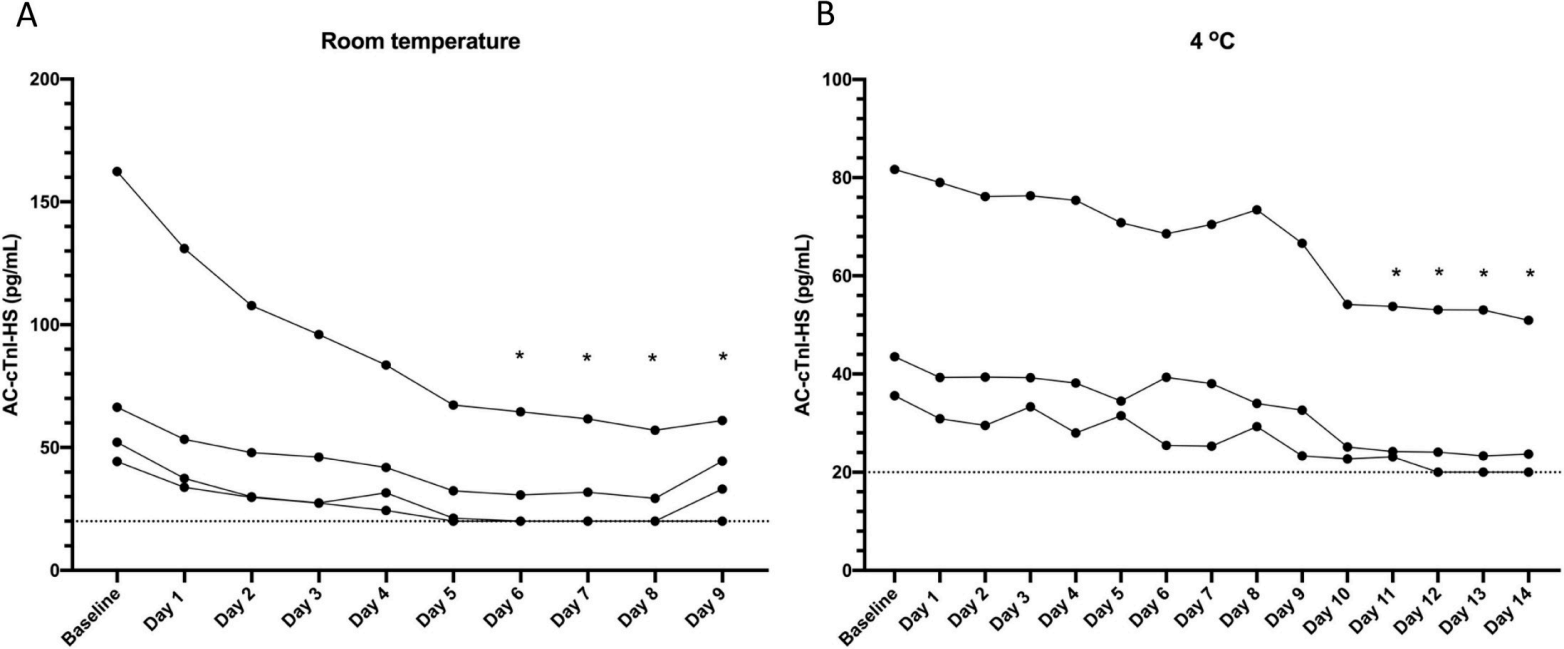
Degradation of cardiac troponin I concentrations at room temperature and 4°C degrees Celsius. Cardiac troponin I measured in serum samples from dogs with the ADVIA Centaur XP High-Sensitivity Troponin I assay are depicted graphically at baseline and daily for 14 consecutive days when stored at room temperature (panel A) or at 4 degrees Celsius (panel B). Due to missing data points (insufficient sample quantity), statistical comparisons could only be made through day six for the room-temperature samples. * indicates a significant difference from baseline.

Degradation of cTnI concentration in canine serum samples stored at room temperature and 4°C are presented in Fig 4. Dogs with AC-cTnI-HS values <20 pg/mL at baseline were excluded, leaving four dogs for comparison at room temperature and three dogs for comparison at 4°C. The concentrations of AC-cTnI-HS were significantly different than the baseline values by day 6 for samples stored at room temperature (P < 0.0064) and by day 11 for samples stored at 4°C (P = 0.048).

**Fig 5:**
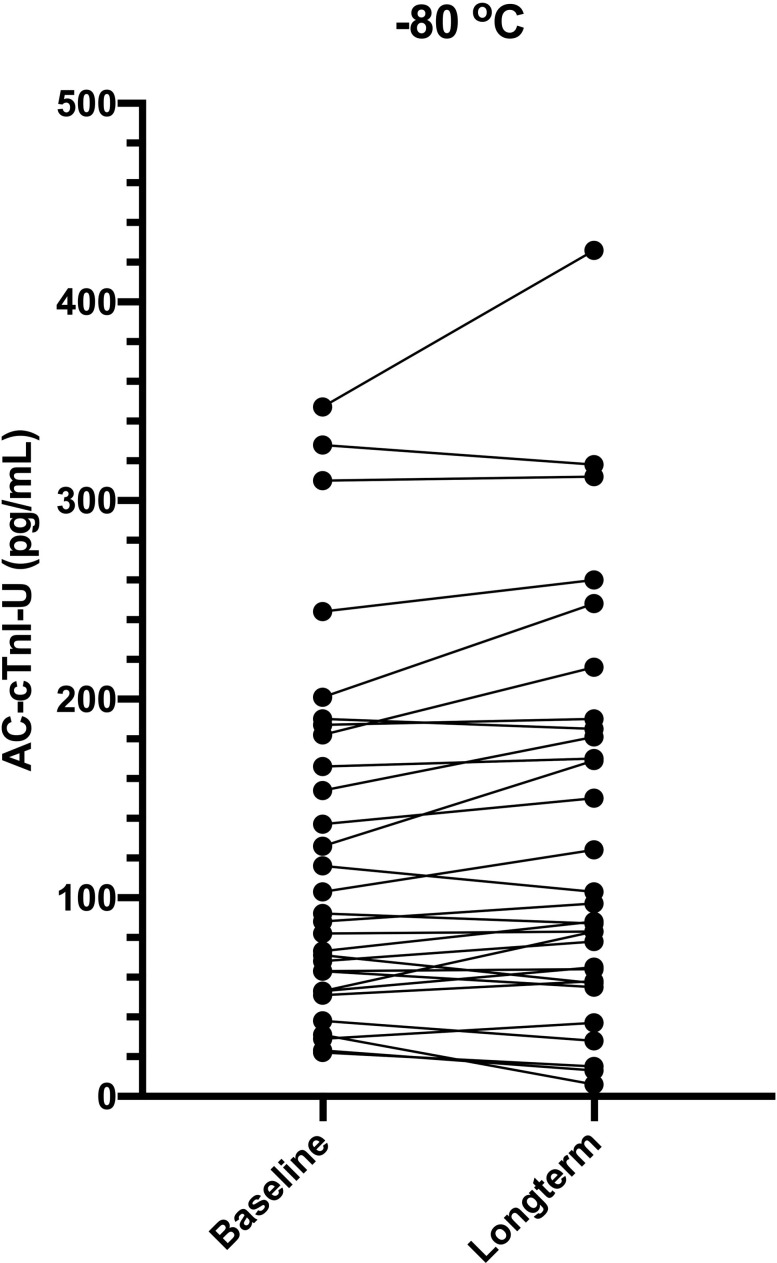
Change in cardiac troponin I concentrations after long-term storage at -80°C. Cardiac troponin I concentrations measured with the ADVIA Centaur TnI-Ultra assay at baseline and after long-term storage at -80°C in 30 dogs are depicted. There was no significant change in values.

Original cTnI concentrations obtained with the AC-cTnI-U assay were compared to repeat measurements using the same assay on the 30 serum sample aliquots that had been frozen at -80°C for 590 days (N = 10), 779 days (N = 10), or 1009 days (N = 10). The cTnI concentration, as measured by the AC-cTnI-U assay, after long-term storage at -80°C was not significantly different from baseline values (P = 0.052). Individual data points are presented graphically in Fig 5. Given the lack of significant difference, the AC-cTnI-HS concentrations measured concurrently from these same 30 samples were included in the clinical population of Group 3 (MMVD).

### Clinical evaluation

The clinical population was comprised of 196 dogs, with 39 in Group 1 (Healthy), 54 in Group 2 (Congenital heart disease), 68 in Group 3 (MMVD), 15 in group 4 (DCM), and 20 in group 5 (Myocarditis). Age, sex, and body weight summary data are presented for each group in Table 4. Breeds represented in Group 1 included Cavalier King Charles spaniels (n = 12), mixed breed dogs (n = 5), Doberman pinschers (n = 3), Borzoi (n = 3), golden retrievers (n = 2), Labrador retrievers (n = 2), and 12 additional breeds represented by one dog each. Half of the dogs in Group 1 were < 6 years of age while half were > 6 years of age. Breeds in Group 2 (i.e., congenital heart disease) included Labrador retrievers (n = 5), mixed breed dogs (n = 5), French bulldogs (n = 4), Pit Bull terriers (n = 4), German shepherds (n = 3), Australian shepherds (n = 3), English bulldogs (n = 2), Yorkshire terriers (n = 2), Border collies (n = 2), Chihuahuas (n = 2), Corgis (n = 2), Fox terriers (n = 2), Golden retrievers (n = 2), and 16 additional breeds represented by one dog each. Diagnoses in Group 2 (congenital heart disease) included patent ductus arteriosus (n = 24), pulmonic stenosis (n = 12), complex congenital disease represented by more than one defect (n = 7), subaortic stenosis (n = 6), and one dog each with a ventricular septal defect, atrial septal defect, tetralogy of Fallot, or mitral valve dysplasia. Breeds represented in Group 3 (MMVD) included Cavalier King Charles spaniels (n = 56), Chihuahuas (n = 5), mixed breed dogs (n = 2), and five additional breeds represented by one dog each. The American College of Veterinary Internal Medicine MMVD stages [28] of dogs in Group 3 were determined to be B1 (n = 43), B2 (n = 20), and C (n = 5). Breeds represented in Group 4 (DCM) included Borzoi (n = 4), Labrador retrievers (n = 2), Whippets (n = 2), and seven additional breeds represented by one dog each. The stage of DCM for dogs in Group 4 was overt (n = 8), occult (n = 6), or equivocal (n = 1). Breeds represented in Group 5 (i.e., myocarditis) included Labrador retrievers (n = 5), Cocker spaniels (n = 5), English pointers (n = 4), German shorthaired pointers (n = 3), Belgian Malinois (n = 2), and a single Pit Bull terrier. All dogs in Group 5 had a positive immunofluorescent antibody titer for *Trypanosoma cruzi*, the etiologic agent that causes Chagas disease.

**Table 4:** Demographic data and serum cardiac troponin I concentrations in healthy dogs and dogs with cardiac disease.

Group	Number	Age (years)	Sex (M/F)	Weight (kg)	AC-cTnI-U (ng/mL)	AC-cTnI-HS (pg/mL)
1 (Healthy)	39	4.3 (0.8–13.7)	19/20	11.6 (2.6–45)	0.028 (0.005–0.393)	22 (<20–229)
2 (Congenital)	54	0.8 (0.2–11.0)	23/31	12.5 (2.8–32.9)	0.026 (0.004–0.326)	25 (<20–236)
3 (MMVD)	68	9.2 (1.1–15.5)	30/38	8.2 (2.8–25.8)	0.083 (0.005–0.518)	55 (<20–351)
4 (DCM)	15	6.1 (1.6–10.2)	11/4	34.6 (12.6–55)	0.413 (0.028–1.861)	255 (24–914)
5 (Myocarditis)	20	5.5 (0.6–9.7)	13/7	21.1 (12.0–30.4)	0.290 (0.106–1.416)	122 (<20–1004)

Demographic data and cardiac troponin I concentrations as measured by the ADVIA Centaur TnI-Ultra assay and the ADVIA Centaur XP High-Sensitivity Troponin I assay are presented across clinical groups. Data are presented as median (range). DCM: dilated cardiomyopathy; MMVD: myxomatous mitral valve disease.

**Fig 6:**
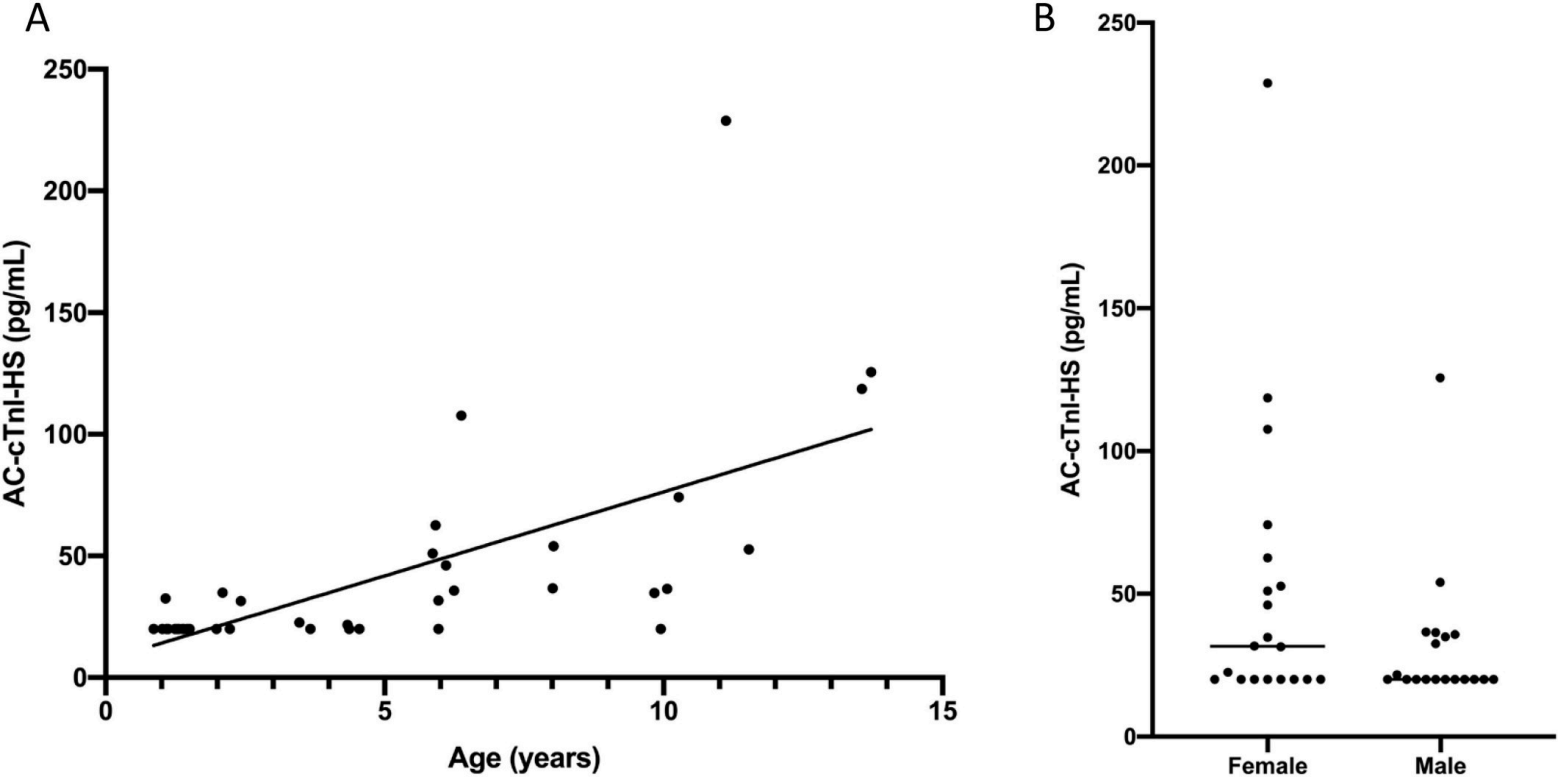
Effect of age and sex on cardiac troponin I concentrations in healthy dogs. The significant positive correlation between age and cardiac troponin I concentrations (r_s_ = 0.80, P < 0.0001) amongst the dogs in Group 1 (Healthy) with the ADVIA Centaur XP High-Sensitivity Troponin I assay is displayed in panel A, while the lack of difference (P = 0.186) between females and males in this group is displayed in panel B.

**Fig 7:**
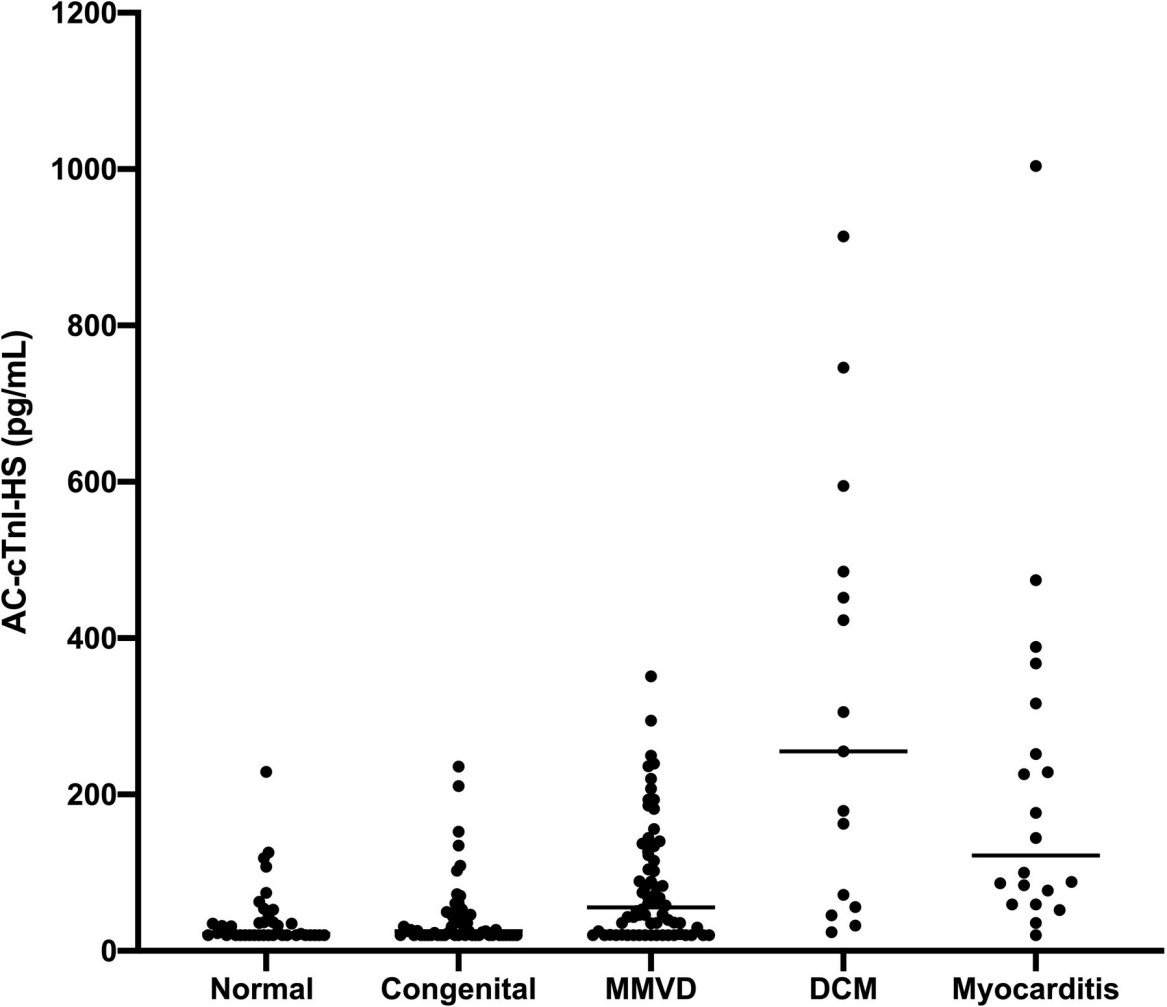
Cardiac troponin I concentrations in healthy dogs and dogs with various cardiac diseases. Cardiac troponin I concentrations measured with the ADVIA Centaur XP High-Sensitivity Troponin I assay in the serum of healthy dogs and dogs with congenital heart disease, myxomatous mitral valve disease (MMVD), dilated cardiomyopathy (DCM), or myocarditis are depicted.

There was a significant positive correlation (r_s_ = 0.747, P < 0.0001) between age and cTnI concentrations amongst the dogs in Group 1 (Healthy) with the AC-cTnI-HS assay, but no significant difference between AC-cTnI-HS concentration in males versus females (P = 0.186) (Fig 6). Serum cTnI concentration was at or below the lower limit of detection of the AC-cTnI-U assay in 38/196 dogs, while only 1/196 dogs had a serum cTnI concentration below the lower 2.5 pg/mL limit of quantification of the AC-cTnI-HS assay in humans. However, 59/196 dogs had AC-cTnI-HS concentrations <20 pg/mL, the lowest repeatably measurable concentration of the assay based on the present inter and intra-assay repeatability data in dogs. Nineteen of 39 dogs (48.7%) in Group 1 (Healthy) had concentrations <20 pg/mL. The concentrations of cTnI as measured by both the AC-cTnI-U and AC-cTnI-HS assays across each group are presented in Table 4 and Fig 7. Group 1 (Healthy) did not show a statistically significant difference in cTnI concentration as measured by the AC-cTnI-HS from that in group 2 (i.e., congenital heart disease) (P > 0.999). However, Group 1 (healthy dogs) did show significantly lower cTnI concentrations than Group 3 (MMVD), Group 4 (DCM), and Group 5 (Myocarditis), with P values of 0.0085, <0.0001, and <0.0001, respectively. Similarly, dogs in Group 2 (i.e., Congenital heart disease) showed significantly lower cTnI concentrations than dogs in Group 3 (MMVD), Group 4 (DCM), or Group 5 (Myocarditis), with P values of 0.0168, <0.0001, and <0.0001, respectively. Dogs in Group 4 (DCM) did not have significantly different serum cTnI concentrations compared to dogs in Group 5 (Myocarditis) (P > 0.999). Dogs in Group 2 were significantly younger than dogs in other groups, while dogs in Group 3 (MMVD) were significantly older than dogs in all other groups with the exception of Group 4 (DCM). With regard to body weight, Group 1 (Healthy dogs) were not significantly different from those in Group 2 (Congenital heart disease) or Group 5 (Myocarditis). However, dogs in Group 3 (MMVD) weighed significantly less than dogs in all other groups, while dogs in Group 4 (DCM) weighed significantly more than dogs in all other groups with the exception of Group 5 (Myocarditis).

## Discussion

The results of this study indicate that the AC-cTnI-HS assay precisely, reproducibly, and accurately measured serum cTnI concentrations in dogs above a concentration of 20 pg/mL. While 99.5% of dogs with and without cardiac disease had measurable cTnI concentrations above the 2.5 pg/mL limit of detection of the AC-cTnI-HS assay established for humans, only 71.4% had measurable cTnI concentrations >20 pg/mL, the lowest concentration with %CV <10% for intra and inter-assay repeatability based on the present canine data. Although there are no generally accepted maximum %CV for intra-assay or inter-assay variability, %CVs of <10% are generally considered to be acceptable [29]. This is compared to 80.6% of dogs having measurable cTnI concentrations using the AC-cTnI-U assay. These findings are discrepant compared to what has been reported for humans, with cTnI concentrations above the limit of detection in 94% of samples using the AC-cTnI-HS assay compared to only 74% of samples using the AC-cTnI-U assay [25]. The lower limit of the blank was also relatively high at 4.5 pg/mL for the zero calibrator and 8.5 pg/mL for the sample diluent compared to what has been reported by the manufacturer (1 pg/mL) [26]. The reason for this difference is not known, though a protein component in the zero calibrator is needed for some assays to perform accurately [30] and may explain the sample diluent lower limit of the blank being higher than the zero calibrator lower limit of the blank for the present data. However, even the zero calibrator limit of the blank was higher than expected and this may have contributed to the poor repeatability and reproducibility for low concentration sample pools. Although the AC-cTnI-HS assay underperforms the previous AC-cTnI-U assay with regard to the lower end of the working range, the assay remains clinically useful, as dogs with overt myocardial injury would be expected to have cTnI concentrations well above 20 pg/mL.

Serial dilutions showed mean O/E values of 125.7% (standard deviation: 34.2%) with a range of 92.3–266.7%. Thus, dilutional linearity for the AC-cTnI-HS assay appeared within the generally acceptable target range of 80 to 120%, especially for expected values >20 pg/mL. This again suggests that there is not a major detrimental matrix effect when measuring cTnI using the manufacturer’s sample diluent. Additionally, the wide working range of the assay suggests that dilution of clinical samples is unlikely to be necessary. The mean O/E ratio for spiking recovery was 95.9% (standard deviation: 9.7%), with a range of 84.2%– 108%. This falls within the generally acceptable range for recovery of 80 to 120%, suggesting the assay is accurate and there is not a detrimental matrix effect. Spiking experiments with purified cTnl were not performed. The AC-cTnI-HS assay, therefore, met the criteria for analytical validation for the measurement of cTnl in dog serum for concentrations > 20 pg/mL. The overall performance characteristics were slightly inferior to those of the AC-cTnI-U assay [7].

Comparison between the AC-cTnI-U and AC-cTnI-HS assays revealed a strong correlation between the two methods, however, a proportional bias was noted whereby the AC-cTnI-HS assay results were proportionally lower than the AC-cTnI-U results, particularly at higher cTnI concentrations. This should be accounted for when interpreting results with the AC-cTnI-HS assay if an attempt is made to compare measurements to previous AC-cTnI-U results. The results of the assays should not, therefore, be used interchangeably. From a clinical perspective, it is important to reemphasize that the AC-cTnI-U is no longer available and therefore the only option is to use the AC-cTnI-HS assay. Additionally, the difference in reported units must be acknowledged, as the AC-cTnI-U assay was reported in ng/mL, while the AC-cTnI-HS assay is reported in pg/mL (or ng/L). The units of the new assay can be converted to the units of the old assay by dividing by 1000.

Sample stability data suggested good long-term stability of serum cTnI at -80°C for up to 2.7 years, as well as adequate sample stability after one freeze-thaw cycle at -80°C. Comparatively, one prior publication assessing cardiac troponin T concentrations in humans showed adequate sample stability at -70°C for up to 12 months, but a significant decrease in sample concentrations at 24 months [31], while another study suggested that cardiac troponin T concentrations in rats were preserved at -80°C for only one week [32]. The present study provides useful data to inform best practices on the storage of canine serum samples intended for cTnI measurement going forward. For clinical samples that must be handled prior to shipping, sample degradation did clearly occur at both room temperature and refrigerator temperature (4°C), with refrigeration resulting in slower decay than storage at room temperature. Ideally, serum samples should be collected and immediately frozen or refrigerated, then shipped on ice to the laboratory for sample analysis for the most accurate results.

The clinical data presented here are largely in agreement with previously published clinical validation data for the AC-cTnI-U assay [6, 7], though it was of interest that the congenital heart disease group did not have significantly higher cTnI values than the healthy population in the present study. Prior clinical data derived using the AC-cTnI-U assay showed that dogs with congenital heart disease, MMVD, and clinically relevant arrhythmias all had significantly higher cTnI concentrations than a healthy dog population [7]. In that study, however, the median age of the healthy population was 2.6 years (compared to 4.3 years in the present study), while the median age of the congenital heart disease population was 5.3 years (compared to 0.8 years in the present study). Given the significant correlation between cTnI concentrations and age, it is possible that the older healthy population and younger congenital population in the present study may have contributed to this difference in results. Additionally, congenital heart disease exists on a spectrum, with substantial heterogeneity in disease severity and expected differences in cTnI concentrations. Differences in the types and severities of underlying congenital heart disease may have resulted in a meaningful difference in the present population compared to prior work.

Also of note, there was not a significant difference between the cTnI concentrations between dogs in Group 4 (DCM) and Group 5 (Myocarditis). While both groups may have variable disease severity and thus variable degrees of serum cTnI elevation, there are also multiple etiologic causes for the development of a DCM phenotype, one of which is myocarditis. Thus, some degree of overlap between these two populations is possible and difficult to tease apart without invasive testing such as collecting an endomyocardial biopsy.

One limitation of the present study is the lack of further stratification within the clinical groups. There were not enough dogs within each group to further subclassify based on MMVD or DCM stage or congenital heart disease diagnosis, thus dogs remained in larger, more heterogeneous groupings. Additionally, renal values and blood pressure measurements were not available for all dogs in the study, therefore an impact of azotemia or systemic hypertension on serum cTnI concentrations could not be considered in this context. Furthermore, the clinically healthy group was defined as such based on clinical history, physical examination, and echocardiogram results. Additional laboratory testing and/or diagnostic imaging was not pursued, thus it is possible that underlying systemic or cardiac disease could have been missed. Lastly, the inter and intra-assay variability measurements were based off of 10 repetitions of each sample. Twenty repetitions would have been ideal.

## Conclusion

In conclusion, the AC-cTnI-HS assay evaluated here precisely, reproducibly, and accurately measures cTnI concentrations in the serum of healthy dogs and dogs with cardiac disease, above a concentration of 20 pg/mL, with 71.4% of studied dogs having a measurable cTnI concentration. The assay is suitable for clinical use in dogs, though it underperforms a previous AC-cTnI-U assay at the lower end of the working range. The AC-cTnI-HS assay correlates well with a previous AC-cTnI-U assay, though a proportional bias exists whereby the AC-cTnI-HS assay measures proportionally lower.

## Supporting information

S1 File Raw data associated with analytical validation of the AC-cTnI-HS assay including inter- and intra-assay variability, dilutional parallelism, spiking recovery, and limit of the blank analysis. (XLSX)

S2 File Raw data associated with sample storage stability. (XLSX)

S3 File Raw data associated with clinical samples in healthy dogs and dogs with cardiac disease. (XLSX)
